# Predicting effects of blood flow rate and size of vessels in a vasculature on hyperthermia treatments using computer simulation

**DOI:** 10.1186/1475-925X-9-18

**Published:** 2010-03-26

**Authors:** Huang-Wen Huang, Tzu-Ching Shih, Chihng-Tsung Liauh

**Affiliations:** 1 Department of Innovative Information and Technology, Software Engineering Group, Langyang Campus, Tamkang University, I-lan County 26247, Taiwan; 2 Department of Biomedical Imaging and Radiological Science, China Medical University and Department of Radiology, China Medical University Hospital, Taichung 40402, Taiwan; 3 Department of Mechanical Engineering, Kun-shan University, Tainan 71003, Taiwan

## Abstract

**Background:**

Pennes Bio Heat Transfer Equation (PBHTE) has been widely used to approximate the overall temperature distribution in tissue using a perfusion parameter term in the equation during hyperthermia treatment. In the similar modeling, effective thermal conductivity (K_eff_) model uses thermal conductivity as a parameter to predict temperatures. However the equations do not describe the thermal contribution of blood vessels. A countercurrent vascular network model which represents a more fundamental approach to modeling temperatures in tissue than do the generally used approximate equations such as the Pennes BHTE or effective thermal conductivity equations was presented in 1996. This type of model is capable of calculating the blood temperature in vessels and describing a vasculature in the tissue regions.

**Methods:**

In this paper, a countercurrent blood vessel network (CBVN) model for calculating tissue temperatures has been developed for studying hyperthermia cancer treatment. We use a systematic approach to reveal the impact of a vasculature of blood vessels against a single vessel which most studies have presented. A vasculature illustrates branching vessels at the periphery of the tumor volume. The general trends present in this vascular model are similar to those shown for physiological systems in Green and Whitmore. The 3-D temperature distributions are obtained by solving the conduction equation in the tissue and the convective energy equation with specified Nusselt number in the vessels.

**Results:**

This paper investigates effects of size of blood vessels in the CBVN model on total absorbed power in the treated region and blood flow rates (or perfusion rate) in the CBVN on temperature distributions during hyperthermia cancer treatment. Also, the same optimized power distribution during hyperthermia treatment is used to illustrate the differences between PBHTE and CBVN models. K_eff _(effective thermal conductivity model) delivers the same difference as compared to the CBVN model. The optimization used here is adjusting power based on the local temperature in the treated region in an attempt to reach the ideal therapeutic temperature of 43°C. The scheme can be used (or adapted) in a non-invasive power supply application such as high-intensity focused ultrasound (HIFU). Results show that, for low perfusion rates in CBVN model vessels, impacts on tissue temperature becomes insignificant. Uniform temperature in the treated region is obtained.

**Conclusion:**

Therefore, any method that could decrease or prevent blood flow rates into the tumorous region is recommended as a pre-process to hyperthermia cancer treatment. Second, the size of vessels in vasculatures does not significantly affect on total power consumption during hyperthermia therapy when the total blood flow rate is constant. It is about 0.8% decreasing in total optimized absorbed power in the heated region as γ (the ratio of diameters of successive vessel generations) increases from 0.6 to 0.7, or from 0.7 to 0.8, or from 0.8 to 0.9. Last, in hyperthermia treatments, when the heated region consists of thermally significant vessels, much of absorbed power is required to heat the region and (provided that finer spatial power deposition exists) to heat vessels which could lead to higher blood temperatures than tissue temperatures when modeled them using PBHTE.

## Background

Hyperthermia is used to raise tissue temperatures in a range of 40-43°C for a long period of time to kill tumorous cells. Within the past 20 years, it has gained much of attention for combining direct thermal toxicity and enhancements of the efficacy of some drugs [1-3]. The application of hyperthermia has been integrated in multimodal treatment strategies in several forms of tumors. Experimental and clinical evidence has indicated that the combination of cytotoxic drugs with localized hyperthermia in the cancer treatment increases the killing of tumor cells [4]. As well as other treatment modalities, they also showed an increasing efficiency in treatments [5].

The Pennes [6] bio-heat transfer equation (PBHTE) has been a standard model for predicting temperature distributions in living tissues for more than half a century now. The equation was established through conducting a sequence of experiments of temperature measurements of tissue and arterial blood temperatures in the resting human forearm. The equation includes a special term that describes the heat exchange between blood flow and solid tissues. The blood temperature is assumed to be constant arterial blood temperature. Some researchers [7-11] also developed alternative equations having the same goal, attempting to formulate a single, general field equation that could predict the overall characteristics of temperature distributions in tissues. For example, K_eff _model (effective thermal conductivity model) [9,11] is a field equation using thermal conductivity as a key parameter to replace blood perfusion term in PBHTE. However, this has been challenged by many research groups internationally when trying to predict temperature distribution in regions which involve isolated large vessels.

Those approximate field equations neither have, nor were they ever intended to have, the ability to accurately model the effects of isolated, large vessels. Such infrequently occurring vessels cannot be simulated by such approximate field equations, which are intended to predict the average thermal behavior of the tissue. Thus, such vessels must be modeled using separate equations. The effect of such vessels have been studied by Chato [12] and Huang et al. [13,14] who developed analytical models for single vessels, and by other investigators [15-23] who have done numerical and experimental hyperthermia studies of single vessels and/or counter current vessel pairs imbedded in either a purely conductive media (with either a normal thermal conductivity, or an enhanced, effective thermal conductivity) or in media modeled by the Pennes BHTE. One of those studies, by Rawnsley et al. [22], compares the predictions from such a combined model (approximate field equation plus a separate blood vessel model) with experimental hyperthermia results. It clearly showed the increased accuracy of such combined models. Leeuwen et al. [23] also stressed that efforts to obtain information on the positions of the large vessels in an individual hyperthermia patient will be rewarded with a more accurate prediction of the temperature distribution. Finally, a few studies have modeled the effect of collections of a large number of parallel vessels or of networks of vessels [23-26].

The present paper describes effects of, blood flow rates and size of vessels in a vasculature, on 3-D temperature distributions and absorbed power distributions using a model [24] comprising a network of blood vessels applied with an optimization to reach the ideal therapeutic temperature distribution of uniform 43°C during hyperthermia cancer treatment. This paper uses a rather simple, generic vessel network model in order to develop and illustrate the basic approach of the thermal model, and to illustrate the types of applications possible for such a model.

## Methods

### Vessel Network Geometry and Fully Conjugated Blood VesselNetwork Model

**Figure 1:**
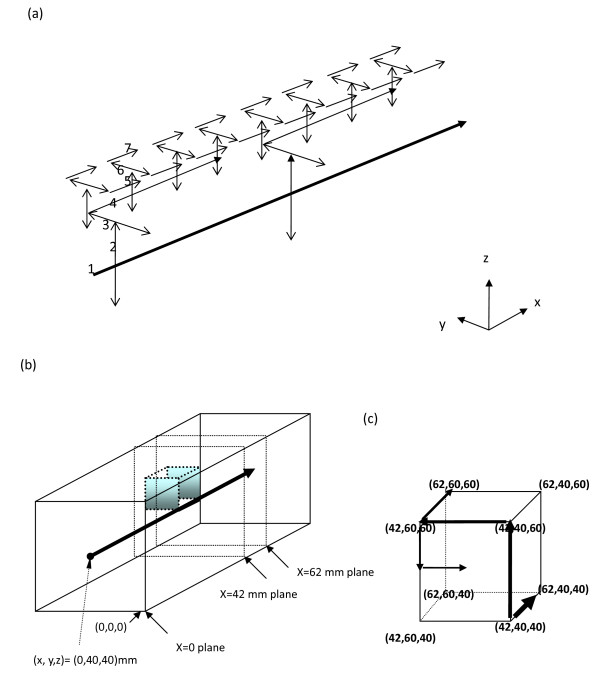
**(a) Schematic of the partial arterial vessel network**. All seven vessel levels (level 1 to level 7) are shown. The venous network is parallel to the arterial network. (b) A transparent view of a parallelepiped showing the internal heated tumor region, which is a cubic volume of 20 mm by 20 mm by 20 mm. The level 1 largest blood vessel is running through the volume's edge from location (42, 40, 40) mm to location (62, 40, 40) mm. The original point is located at the southeastern corner when facing the x = 0 plane. (c) The locations of the cubic volume in a parallelepiped by indicating its 8 corners' coordinate. The units are millimeters (mm).

The geometry used consists of a regular, branching vessel network, as partially shown in (only schematic of the partial arterial vessel network) Figure 1(a), that is embedded in a control volume which is an (approximate) cube of dimensions *L *= 82 mm and *W *= *H *= 80 mm in the *x, y*, and *z *directions, respectively. All seven vessel levels (level 1 to level 7) are shown. The venous network is parallel to the arterial network. All vessels are straight-line segments parallel to one of the three Cartesian axes. There are up to seven levels of arteries, beginning with the main artery (level one) which lies along the central, lengthwise (*x*) axis of the cube. Table one of [24] listed the basic vessel network properties used in these studies. The diameters of the arteries decrease by a constant ratio γ between successive levels of branched vessels (the ratio of diameters of successive vessel generations) i.e.(1)

where *D*_*i *_and *D*_*i*+1 _are the diameters of two successive levels of branching arteries.

When two successive levels of numbered vessels do not branch but only change direction (i.e., levels six and seven in this model) the vessel diameter does not change. Figure 1 (b) shows a transparent view of a parallelepiped indicating internal heated tumor region (or cell) and the level 1 artery running downstream from (0, 40, 40) mm (at center of inlet boundary plane) to (42, 40, 40) mm which is a branching location point from level 1 artery vessel to level 2 artery vessels (along z-axis). The units of coordinates are mm. The location point, (42, 40, 40), is also a corner of heated tumor region (or cell) as an enlarged view shown in Figure 1(c). The tumor has a cubic volume of 20 mm by 20 mm by 20 mm, which volume is illustrated by its 8 corner locations. A passing level 1 artery vessel (carries with 0.1% of whole control volume blood perfusion) is shown, in Figures 1(a) and 1(b), from the branching location (42, 40, 40) to the exit point (82, 40, 40) of outlet boundary plane. Thus the passing level 1 artery contributes rather insignificant heat exchange with surrounding tissues. Figure 1(c) also shows the pathway of partial arterial branching vessels on the margin of the tumor and the pathway consists of the level 1 vessel segment (from (42,40,40) to (62,40,40)), the level 2 vessel segment (from (42,40,40) to 42,40,60)), the level 3 vessel segment (from (42,40,60) to (42,60,60)), the level 4 vessel segment (from (42,60,60) to (62,60,60)), the level 5 vessel segment (from (42,60,60) to (42,60,50)) and the level 6 vessel segment (from (42,60,50) to (42,50,50)). The locations (42, 60, 50) and (42, 50, 50) are not corners of the heated tumor volume and they are not shown in Figure 1(c). The level 1 vessel branching point at (42, 40, 40) and all its subsequent branching vessels to lower level vessels present identical pattern for the branching point at (2, 40, 40) and all its subsequent branching vessels to lower level vessels. In other words, the tumor cell resides on the location ranging from (42 to 62, 40 to 60, 40 to 60) of (x, y, z) coordinates and all other vessels (arterial part) around the cell are highlighted in Figure 1(a).

The geometric arrangement of the counter current veins is essentially identical to that of the arteries, with all of the veins offset from the arteries by one finite difference node in x, y, and z as appropriate to avoid intersections of vessels. Each of terminal arteries is assumed to supply all of the blood to a defined subvolume of tissue and tumor regions which subvolume was defined according to Figure 2(a) of Huang et al [24].

**Figure 2:**
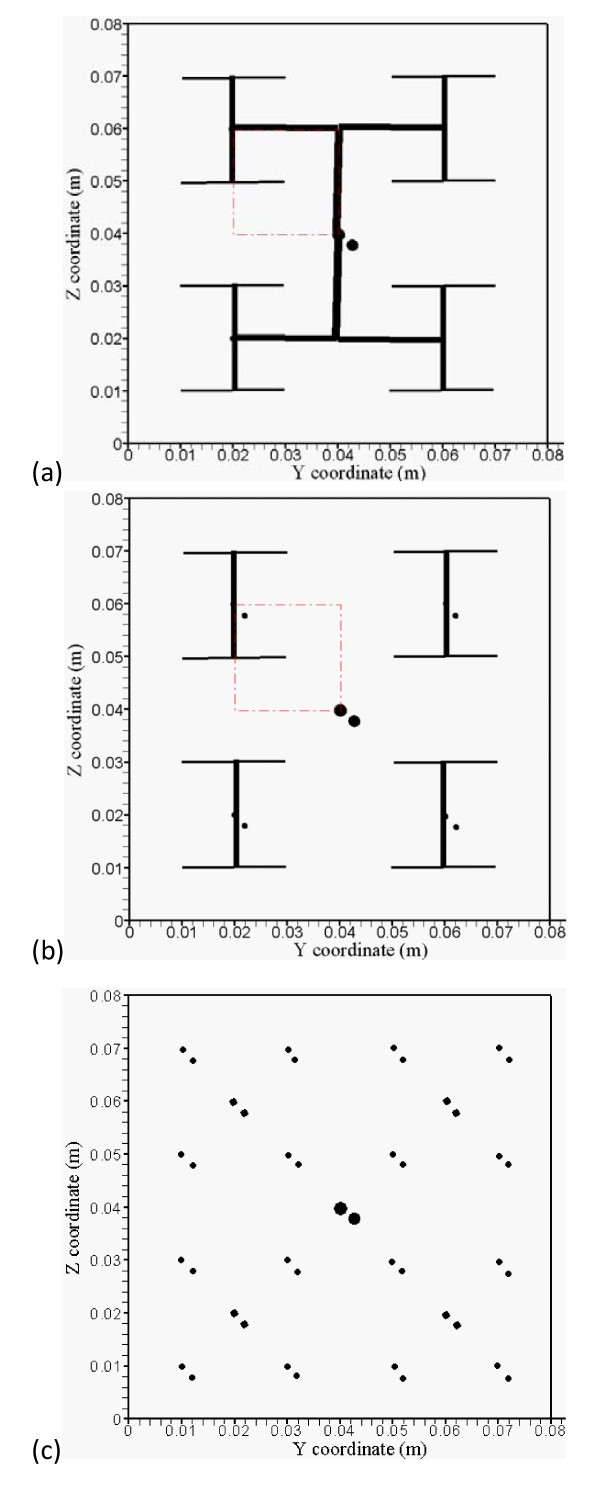
**(a) The branching vessels at the x = 42 mm cross-section plane**. Red dash-dot square lines indicate the heated region. (b) The branching vessels at the x = 52 mm and 62 mm cross-section planes. Red dash-dot square lines indicate the heated region. (c) The branching countercurrent artery-vein vessels run in x direction at the x = 38 mm and 66 mm cross-section planes.

To better comprehend the results of temperature and optimized power fields with complicated branching artery and vein vessels, five detailed vasculature cross-section planes are shown in Figures 2(a)-2(c) to illustrate countercurrent artery-vein vessels pathways and locations. Figure 2(a) shows arterial branching vessels at the x = 42 mm cross-section plane. Red dash-dot square lines indicate the heated region, and Figure 2(b) shows arterial branching vessels at the x = 52 mm and 62 mm cross-section planes. Figure 2(c) shows branching countercurrent artery-vein pair vessels running in the x direction at the x = 38 mm and 66 mm cross-section planes which are not in the heated region.

CBVN is a fully conjugated blood vessel network model formulation which describes the solid tissue matrix having thermally significant vessel generations (seven levels). The effects of all vessels smaller than the terminal (level seven) vessels are not explicitly modeled in CBVN. Thus, those smaller vessels (connected to the terminal arteries and the terminal veins in the network) are implicitly assumed to be thermally insignificant in the CBVN. Details of the model were described in Huang et al [24].

### Mathematical equations for the thermal model

The governing equations for both tissues and vessels are described below, the PBHTE for calculating tissue temperatures is:(2)

where *k, c*_*b*_, *w*_*b*_, and *q*_*s *_are thermal conductivity of soft tissue, specific heat of blood, blood perfusion rate and absorbed thermal power density, respectively. The metabolism effect is neglected in Eqn. (2) due to its limited effect on temperature distribution during hyperthermia. The convective energy equation is solved for blood temperatures of vessels in the CBVN model. That is:(3)

where *m*_*b, i *_is the blood mass flow rate at the level i vessel segment and s_*i *_is a coordinate along the axis of the level i vessel segment. *Nu, k*_*b*_, *R*_*bv, i *_and *T*_*w *_are Nusselt number, thermal conductivity in blood, radius of blood vessel at level i and blood vessel wall temperature, respectively. A total of 682 vessels in the model need to be calculated using Eqn. (3). Special treatments of perfusion and collecting blood (to and from subvolumes) on terminal ends (arteries and veins) are added to Eqn. (3) for level 7 vessel calculations. In all cases studied in the present paper, the true tissue perfusion () was assumed to be uniform everywhere, i.e.,  for *I *= 1-128 (the subscript *tsv *indicates terminal subvolume in the control volume). It is the same blood perfusion unit as *w*_*b *_described in PBHTE.

Additionally, a simple K_eff _model is described in Eqn. (4). That is,(4)

where K_eff _is an effective conductivity tenor, T is temperature field and q_*s *_is absorbed thermal power.

### Optimization scheme

The procedure to continuously adjust power deposition in hyperthermia cancer treatment in order to reach ideal temperature (uniform temperature of 43°C throughout the volume of the tumorous region) is described below.

1. Set initial power field equal to 10^5 ^W/m^3 ^uniform in the treated region.

2. Solve governing equations in tissue and blood temperature distributions with given boundary conditions and inlet temperatures of vessels, which are all set to be 37°C.

3. Compare predicted temperature field with ideal temperature field (which is uniform temperature throughout the tumor volume) and calculate criteria value expressed below in Eqn. (5).

4. If criterion value does not meet the condition described in Eqn. (5), power is updated according local temperature as it is described below in Eqn. (6). Go to step 2 and continue the loop.

5. If the criterion meets the condition, the optimal power and temperature distributions are obtained.

The criterion of power deposition theoretically is described in Eq. (5), which states that the root mean square of difference of ideal temperature (which is set to be 43°C) and calculated temperature in treated region of all heated target nodes divided by (43-37)°C reaches less than the criterion value (is set to be 10% of the temperature difference of (43-37)°C). If the criterion is met, we have obtained the optimization of absorbed power such that the estimated temperature distribution is close to the ideal temperature distribution. Otherwise, power deposition will be adjusted according to local temperature, i.e. it is a function of position and temperature. The readjusted power deposition (*P*_*n *+ 1_) is described in Eqn. (6). Its unit is the same as *q*_*s *_which are used in Eqns. (2), (3) and (4).(5)

where Δ *p*(*x*, *y*, *z*) = Coef · Δ*T*(*x*, *y*, *z*), *Coef *is 10000, n is the iteration number and Δ*T(x, y, z) *is the difference of ideal temperature (43°C) and calculated temperature. Smaller Coef values cause more repetitive loops in adjusting power deposition field to reach ideal temperature distribution. In other words, much more time is required to process the optimization. If the power deposited on a site that causes temperature in tissue raised over 43°C, the power will readjust its power deposition to a smaller one in the scheme for an ideal temperature distribution.

### Numerical Methods

The numerical scheme used to calculate the temperatures was a black and red finite difference SOR method [27], with upwind differencing used for the vessels. The numerical details are described by Chen [28] and Huang [14]. Special algorithms were used to account for the vessel corners where arteries and veins change direction, and where two or more arteries divide, or two or more veins join. The thermal resistances around the circular vessels were calculated using the logarithmic resistance approach as described by Chen and Roemer [29]. The property values used in treated tumorous and non-treated normal tissues were *k*_*t *_= 0.5 *W/m^3^/°C*, c = *c*_*b *_= 4000 *J/kg/°C *and ρ = 1000 kg/*m*^3^. The vessel heat transfer coefficient (*h*) was calculated using a constant Nusselt number of four (4) for all vessel levels. In all cases, a finite difference nodal spacing of 2 mm was used. Test results with a nodal spacing of 1 mm for test cases using either the arterial vessel network (when no veins are present) or the counter current vessel network showed no significant differences with the results of the comparable 2-mm nodal spacing models. This 2-mm spacing gives an inter-vessel centerline-to-centerline diagonal spacing of 2.8 mm for the counter current vessels due to the 2-mm offsets in *x*, *y*, and *z*. The blood temperature in vessels was calculated using a steady uniform velocity profile for blood flow that has been used widely and accepted in many investigations. Recently Horng et al [30] reconfirmed it. The boundary condition for temperature in the control volume is 37°C for all surfaces of the parallelepiped. So are the inlet temperatures for level 1 artery and vein.

### Results

Testing of this vascular network model has been done by several means [14] including: a check of the single-vessel numerical results (all vessels removed except the level one artery) against an analytical solution; a verification that all temperatures vary linearly with the applied power; and a check that for all results presented in this paper that the overall energy balance on the control volume was accurate to within 0.1 percent. Figure 3 of [24] shows the vessel diameters, total surface areas, and velocity distributions (for  = 0.5) as a function of the vessel level. The general trends present in that figure are similar to those shown for physiological systems in Green [31] and Whitmore [32].

**Figure 3:**
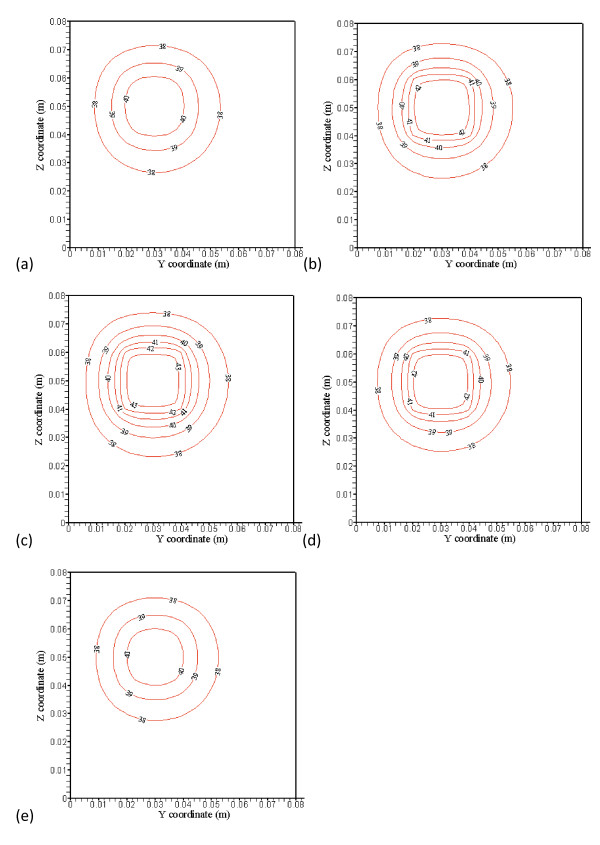
**(a), (b), (c), (d) and (e) show no blood vessel structure present and temperature distributions at the x = 38 mm (4 mm away from the front boundary), x = 42 mm (the front boundary), x = 52 mm (middle of the treated region), x = 62 mm (the back boundary), and x = 66 mm (4 mm away from the back boundary) planes, respectively, with perfusion rate of 0.5 kg· m^-3^s^-1 ^and optimized power shown in Figure 4(a), (b), (c), (d) and (e)**.

**Figure 4:**
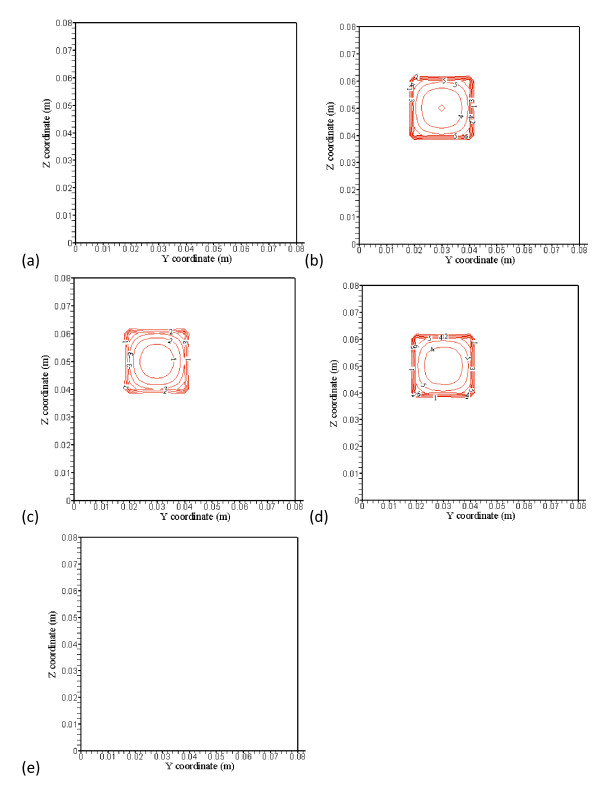
**(a), (b), (c), (d) and (e) showed optimized power distributions at the corresponding plane locations of Figure3**. No power is used in Figs. 4(a) and (e), which are outside of the treated zone. There are, respectively, 6 levels of iso-temperature and iso-power contours, shown in Figures 3, 4, 5 and 6. From low to high (six) levels, the temperature (C) set is {38, 39, 40, 41, 42 and 43} and the power (Wm^-3^) set is {30,000, 60,000, 90,000, 120,000, 150,000 and 180,000}.

**Figure 5:**
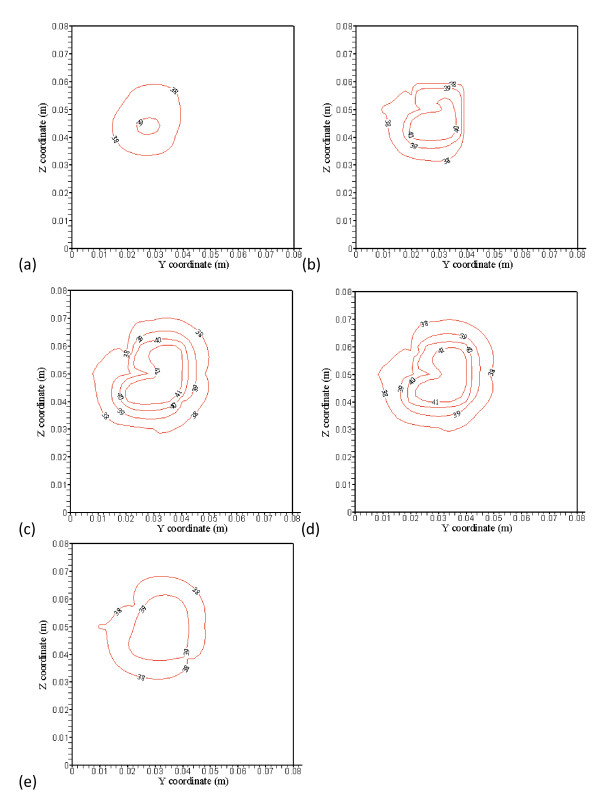
**(a), (b), (c), (d) and (e) show with blood vessel structure present and temperature distributions at the x = 38 mm (4 mm away from the front boundary), x = 42 mm (the front boundary), x = 52 mm (middle of the treated region), x = 62 mm (the back boundary), and x = 66 mm (4 mm away from the back boundary) planes, respectively, with perfusion rate of 0.5 kg· m^-3^s^-1^**. The applied absorbed power distributions are the identical optimized power distributions shown in Figs. 4(a), (b), (c), (d) and (e) for the case of PBHTE.

**Figure 6:**
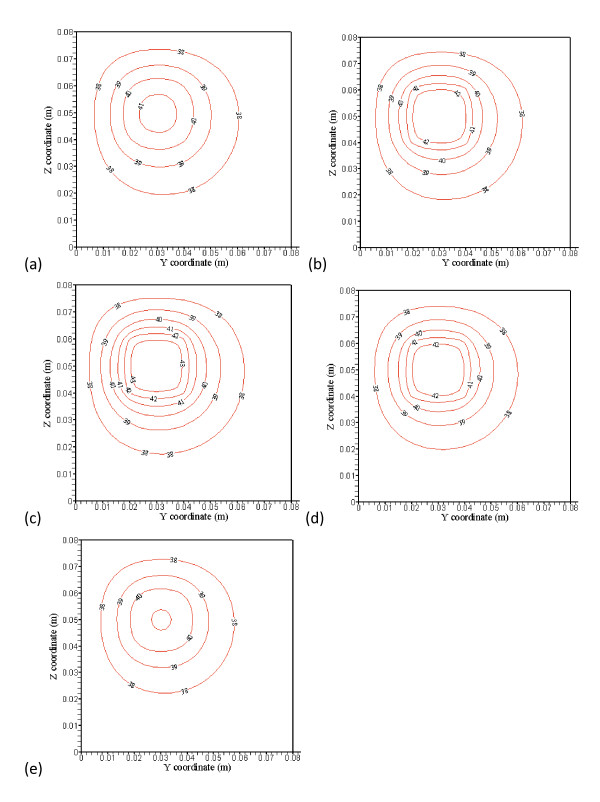
**(a), (b), (c), (d) and (e) showed temperature distributions using a simple K_eff_model at the corresponding plane locations with optimal absorbed power**. There are, respectively, 6 levels of same iso-temperature and iso-power contours shown in Figures 3, 4, 5 and 6.

Figures 3(a), (b), (c), (d) and 3(e) show typical temperature predictions using PBHTE. That is, no blood vessels structure present and temperature distributions at the *x *= 38 mm (4 mm away from the front boundary), *x *= 42 mm (the front boundary), *x *= 52 mm (middle of the treated region), *x *= 62 mm (the back boundary), and *x*= 66 mm (4 mm away from the back boundary) planes, respectively, with perfusion rate of 0.5 kg· m^-3^s^-1 ^and optimized power shown in Figures 4(a), (b), (c), (d) and 4(e). No power is used on Figures 4(a) and 4(e), which are outside of the treated zone. There are, respectively, 6 levels of iso-temperature and iso-power contours, shown in Figures 3, 4, 5 and 6. From low to high (six) levels, the temperature (°C) set is 38, 39, 40, 41, 42 and 43} and the power (Wm^-3^) set is {30,000, 60,000, 90,000, 120,000, 150,000 and 180,000.

The treated cubic volume (20 mm by 20 mm by 20 mm) is applied with the optimized power. Temperature distributions inside the treated region illustrated in Figures 3(b), (c) and 3(d) show very uniform temperature fields, with an average temperature of about 43°C. Temperature distributions in Figures 3(a) and 3(e) at the plane 4 mm away from the boundary of the treated region show maximum temperatures of about 41°C and 40.8°C, respectively. It is about 2°C difference from ideal therapeutic temperature. The temperature distributions are calculated by PBHTE with a perfusion rate of 0.5 kg· m^-3^s^-1^. Figure 4(b) shows optimized power at the front boundary plane. Much of the power is focused on the corners and edges of the treated region to compensate for thermal energy loss through conduction. Figure 4(d) shows optimized power at the back boundary plane. It indicates a power pattern identical with that shown in Figure 4(b). The optimized power in the middle plane of the treated region (Figure 4(c)) shows relatively less power deposited on corners and center area, as compared to the boundary planes. It illustrates the thermal diffusion rate is relatively small at this plane. The ideal temperature is set to be 43°C. Figures 4(a) and 4(e) indicate no power absorbed at the two planes.

Figures 5(a), (b), (c), (d) and 5(e) show temperature predictions using CBVN. That is, with blood vessels structure present and temperature distributions at the *x *= 38 mm (4 mm away from the front boundary), *x *= 42 mm (the front boundary), *x *= 52 mm (middle of the treated region), *x *= 62 mm (the back boundary), and *x*= 66 mm (4 mm away from the back boundary) planes, respectively, with perfusion rate of 0.5 kg· m^-3^s^-1 ^and applied identical absorbed power shown in Figures 4(a), (b), (c), (d) and 4(e). To illustrate the discrepancy of the three models (PBHTE, CBVN and K_eff_), the temperature prediction at those identical locations using a simple Keff model with optimized absorbed power (similar to those graphs shown in Figures 4(a), (b), (c), (d) and 4(e)) are shown in Figures 6(a), (b), (c), (d) and 6(e). In present study, a simple scalar K_eff _(effective conductivity model) is used. The value of effective conductivity is 0.5 W/m^3^/°C which is the same as thermal conductivity of tissue (K_eff_/K_*t *_= 1), and it also is the same as thermal conductivity of blood.

With the applied optimized absorbed power distributions for the case of PBHTE model (without vessels structure) identical to those in the case of CBVN (with vessels structure present), temperature distributions in Figures 5(a) and 5(e) at the plane 4 mm away from the boundary of the treated region show maximum temperatures of about 39°C and 39.6°C, respectively. It is about 4°C different from ideal therapeutic temperature. Figures 5(b), (c) and 5(d) show maximum temperatures of about 40.9, 41.3 and 41.3°C, respectively. An obvious cooling effect from the vasculature appears on the figures. From the temperature distributions, irregular iso-temperature contours indicate significant impact by blood vessels. Cold spots and significant cooling effects of mass flow rate by vessels at the periphery of the tumor volume present vital characteristics in the model. These are consistent with many clinical and experimental phenomena. Unsuccessful hyperthermia treatments lead to survival of cancerous tissues. Insufficient net absorbed thermal energy in localized tissue area is one of the major problems. Figures 6(a), (b), (c), (d) and 6(e) display similar temperature distributions of PBHTE using K_eff _model approach which shows a uniform and smooth temperature field.

**Figure 7:**
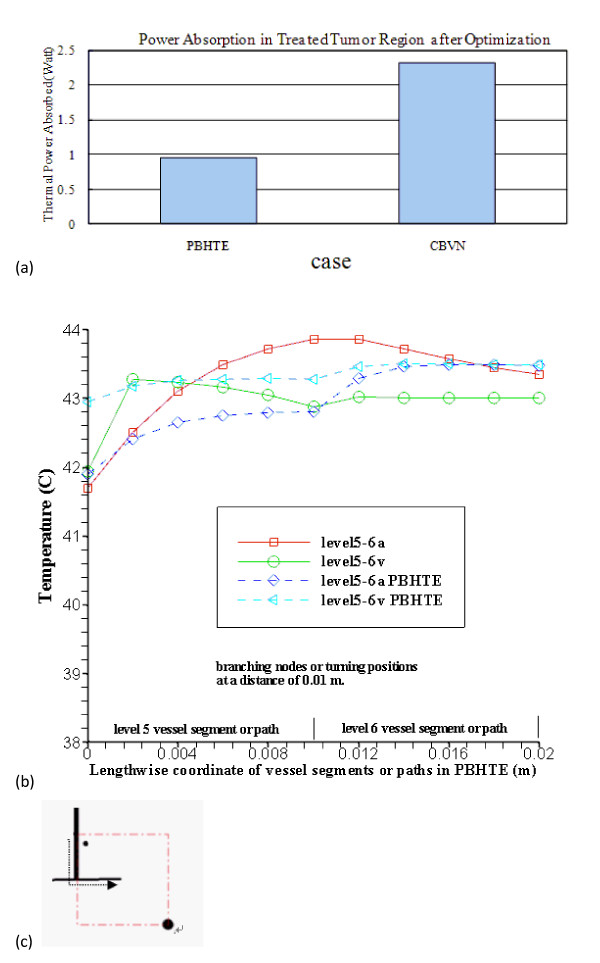
**(a) Case Comparison for Total Power Absorption in Treated Tumor Region after Optimization**. PBHTE is the case when no blood vessel presents. And the case of CBVN has the same blood perfusion rate as the PBHTE case but includes a network of vessels which reveal blood flow rate about 320 mm/sec in level 1 artery and vein vessels. The perfusion rate of 0.5 kg/(m^3^s) in tissues which are perfused by blood vessels is used for PBHTE. 7(b) The comparison of temperatures along level 5 and level 6 vessel segments after optimization at middle plane of the heated region (arterial route: from 0 to 0.02 m; veinous route: from 0.02 to 0) in case of CBVN model (level5-6a represents arterial path consists of level 5 and level 6 vessels; level5-6v represents veinous path consists of level 5 and level 6 vessels) and those temperatures in case of PBHTE model (level5-6a PBHTE represents those arterial path locations which are calculated via PBHTE instead of CBVN; level5-6v PBHTE represents those veinous path locations which are calculated via PBHTE instead of CBVN). 7(c) Lengthwise coordinate (indicated by a dash line) from the intersection of level 3-4-5 vessels to the level 6-7 intersection at the middle plane of heated region shown in Fig. 2(a)

Figure 7(a) shows more than double the power is needed for the CBVN model as compared to PBHTE model for the total power absorption in treated tumor region after optimization. PBHTE is the case with no blood vessels present. Both cases have the same blood perfusion rate, however CBVN presents a vasculature that carries blood flow from a main vessel (level 1) into branching vessels and, at the terminal ends, perfused blood to tissues. Therefore, a blood flow rate of about 320 mm/sec in level 1 artery and vein vessels enables the blood to perfuse into tissues with a rate of 0.5 kg· m^-3^s^-1^. The perfusion rate of 0.5 kg· m^-3^s^-1 ^in tissues is a perfusion term parameter which is used for PBHTE. Figure 7(b) shows the comparison of optimal temperatures along level 5 and level 6 vessel segments at middle plane of the heated region (arterial route: from 0 to 0.02 m; veinous route: from 0.02 to 0) in case of CBVN model (level5-6a represents arterial path consists of level 5 and level 6 vessels; level5-6v represents veinous path consists of level 5 and level 6 vessels) and those temperatures in case of PBHTE model (level5-6a PBHTE represents those arterial path locations which are calculated via PBHTE instead of CBVN; level5-6v PBHTE represents those veinous path locations which are calculated via PBHTE instead of CBVN). Figure 7(c) shows lengthwise axis (indicated by a dash line) from the intersection of level 3-4-5 vessels to the level 6-7 intersection at the middle plane of heated region shown in Figure 2(a).

**Figure 8:**
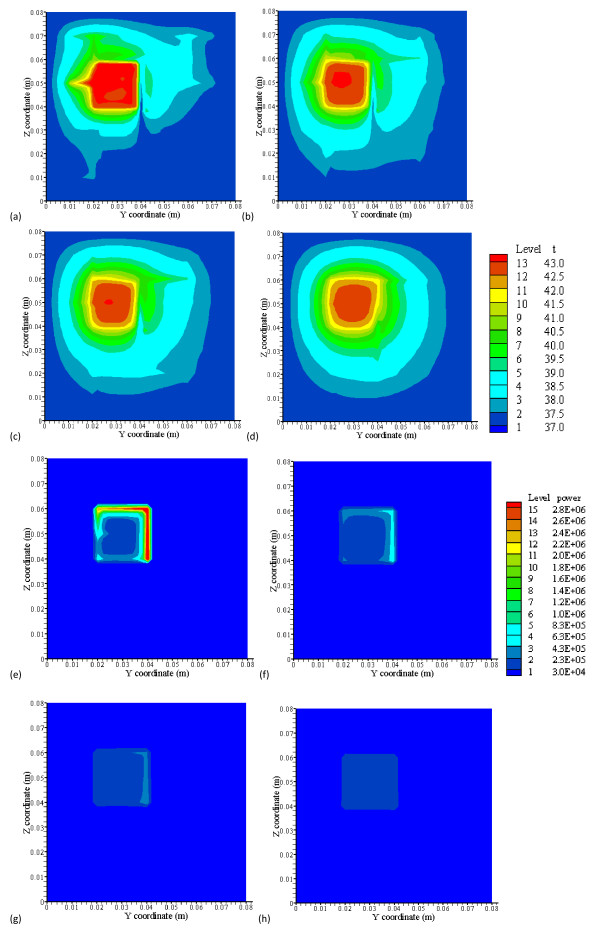
**(a), (b), (c) and (d) show the temperature distributions at the x = 42 mm, the front boundary plane (the level 2 branching vessel plane), for low perfusion rates of 0.5, 0.1, 0.05 and 0.01 kg· m^-3^s^-1^, respectively**. (e), (f), (g) and (h) show their optimized absorbed power distributions, respectively. From low to high levels, the temperature (C) 13-level set is {37, 37.5, 38, 38.5, 39, 39.5, 40, 40.5, 41, 41.5, 42, 42.5 and 43} and the power (Wm^-3^) 15-level set is {3.0e4, 2.3e5, 4.3e5, 6.3e5, 8.3e5, 1.0e6, 1.2e6, 1.4e6, 1.6e6, 1.8e6, 2.0e6, 2.2e6, 2.4e6, 2.6e6 and 2.8e6}.

Figure 8 shows (a), (b), (c) and (d), the temperature distributions by CBVN at x = 42 mm, the front boundary plane (Figure 2(a)), for low perfusion rates of 0.5, 0.1, 0.05 and 0.01 kg· m^-3^s^-1^, respectively. Figures 8(e), (f), (g) and 8(h) are their optimized absorbed power distributions, respectively. Higher magnitudes of absorbed power are located on or near vessels. As perfusion decreases, magnitude of optimized power on vessels decreases. Figure 8(h) shows very small and uniform power distribution in the heated region as perfusion reaches 0.01 kg· m^-3^s^-1^. As shown in Figures 8(f) and 8(g), an increase of perfusion rate in the heated region makes level 2 vessels significant, because vessels gain higher blood speeds and require more power to heat the fluid. On the other hand, strong temperature perturbation caused by vessels' convection effect has significantly dampened as blood perfusion decreases. In Figure 8, the legend from low to high levels, the temperature (°C) 13-level set is 37, 37.5, 38, 38.5, 39, 39.5, 40, 40.5, 41, 41.5, 42, 42.5 and 43} and the power (Wm^-3^) 15-level set is {3.0e4, 2.3e5, 4.3e5, 6.3e5, 8.3e5, 1.0e6, 1.2e6, 1.4e6, 1.6e6, 1.8e6, 2.0e6, 2.2e6, 2.4e6, 2.6e6 and 2.8e6}.

**Figure 9:**
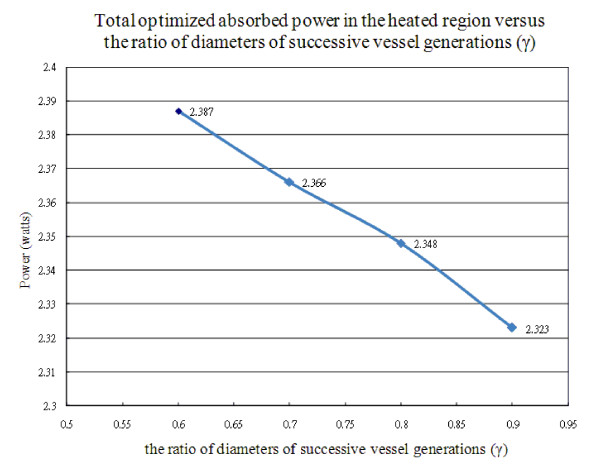
**Total optimized absorbed power in the heated region versus the ratio of diameters of successive vessel generations (γ) in the vasculature, when the region's perfusion rate is 0.5 kg· m^-3^s^-1^**.

Figure 9 shows total optimized absorbed power in the heated region versus the ratio of diameters of successive vessel generations (γ) in the vasculature of the region with perfusion rate of 0.5 kg· m^-3^s^-1^. As γ increases from 0.6 to 0.7, it indicates diameters of successive vessel generations are bigger. The total power required to heat the tumor volume decreases from 2.387 to 2.366. This is about 0.8% decrease in total optimized absorbed power in the region. There is approximately the same trend for γ that increases from 0.7 to 0.8, and from 0.8 to 0.9.

## Discussion

As PBHTE has been widely used in predicting overall temperature behaviors in tissue for many studies, mistaken prediction occurs, when in practicality, a vasculature with thermally significant vessels exists in or near the treated region. As shown in Figures 3 and 4, considering the treated region to be homogenous and without any vascular effect, we modeled it as a PBHTE (or K_eff _model) with some perfusion rate parameter. We then obtained the prediction of optimal absorbed power field for the assumed treated region. However, if in fact the region has a vasculature with thermally significant vessels, we clinically or experimentally applied the predicted optimal power field in the treated region. Therefore undercooling effect by vessels to the ideal temperature results as illustrated in Figure 5(a). In present results, cold strips, heterogeneous temperature spots and significant cooling effects by vessels in the treated region present vital characteristics in the CBVN model. These phenomena reveal the similar critical situations during treatments. Mostly, unsuccessful hyperthermia treatments lead to survival of cancerous tissues. Thus, insufficient net absorbed thermal energy in localized tissue area is one of the major problems. Furthermore, the presented results indicated an ideal case of complete absorbed thermal power in blood which usually generates attenuation in absorbing power (e. g. ultrasound power).

Figure 7(a) shows that the difference in total optimized absorbed power between two models: a traditional bio-heat transfer equation (PBHTE) and the CBVN model. The CBVN model requires more absorbed power to heat the blood fluid in the treatment to reach the ideal therapeutic temperature due to vessels entering with lower inlet (to treated volume) temperatures. The required absorbed power is about twice of the value of that in the PBTHE case. To reveal detailed temperature discrepancy in the two models, the lengthwise vessel locations containing level 5 and level 6 vessel segments at middle plane of heated region are chosen to compare those locations when using PBHTE model, that is shown in Figure 7(b). All temperatures in both models after optimization are in the range of 42-44°C. In CBVN model, blood (i.e. in arterial vessel) has higher temperatures than those (i.e. tissue) locations when using PBHTE in some locations, since blood has gained significant absorbed power during treatment. On the other hand, veinous case does not have large temperature difference than arterial one. One of reasons is that veinous vessels collecting blood from surrounding tissue temperatures which were well heated under treatment.

Additionally from Figures 8(e) and 8(f), they indicated that much of the power absorbed in the treated region after optimization is focused on (or near) blood vessels or dense vessel area. This power optimization scheme offers finer spatial power resolution. A non-invasive heat transducer such as high-intensity focused ultrasound (HIFU) has great potential to perform the optimization scheme. Therefore, uniform ideal temperature is possibly reached throughout the treated region except for some cold spots due to significant vessels near the tumor boundary.

As the perfusion in the treated region decreases, the hyperthermia treatment improves significantly, as shown in the temperature distributions (Figures 8(a)-(d)). Temperature fields show more uniformity in the region as blood flow rate becomes smaller. That is, less temperature perturbation caused by vessels. These results show a systematic approach to reveal the impact of a vasculature of blood vessels against a single vessel which most studies have presented. Cold strips and cold spots are significantly reduced and/or disappear. Given those low perfusion rates of 0.5, 0.1, 0.05 and 0.01 kg· m^-3^s^-1 ^(Figure 8(a)-(d)) in the region with a vasculature, the blood flow speeds of level 1 branching vessel are calculated, and they are approximately 320, 64, 32 and 6 mm/s for those perfusion rates, respectively. This result suggests that reducing or preventing blood flow rate flowing into vasculature(s) of the tumorous treated region is helpful to the treatment. The magnitude of power is reduced as well when perfusion rate decreases.

As the results show in Figure 9, total optimized absorbed power in the heated region does not significantly change total power as the ratio of diameters of successive vessel generations increases from 0.6 to 0.9. Every increment of 0.1 of γ in the range of 0.6 to 0.9 only causes about 0.8% decrease of total absorbed power. Thus, it suggests the size of vessels in vasculature does not significantly affect total power consumption during treatments provided that constant perfusion rate at the region. For example, several thermally significant vessels and their blood flow rates are identified in the treated region; the size of diameters of vessels does not affect much the total absorbed power in the treatment thereafter. This helps in simplifying hyperthermia treatment process once the locations and blood flow rates of thermally significant vessels are identified.

## Conclusion

In summary, these studies suggest that first, any medical strategy that could decrease or prevent blood flow rates into the tumorous region is recommended as a pre-process to hyperthermia cancer treatment or thermal surgery. Second, the size of vessels in vasculatures does not significantly affect on total power consumption during hyperthermia therapy when the total blood flow rate is constant. Also, for the presence of thermally significant vessels in the treated region, particularly with high perfusion rate (i.e. greater than 0.5 kg· m^-3^s^-1^), PBHTE is not a suitable model to predict the temperature distribution. Last, in hyperthermia treatments, when the heated region consists of thermally significant vessels, much of absorbed power is required to heat the region and (provided that finer spatial power deposition exists) to heat vessels which could lead to higher blood temperatures than tissue temperatures when modeled them using PBHTE.

Future efforts should be aimed at developing more accurate and tissue-specific fully conjugated models which can better predict actual tissue temperatures in in-vivo situations.

## Competing interests

The authors declare that they have no competing interests.

## Authors' contributions

HWH developed the idea of an optimization scheme of temperature distributions during hyperthermia treatments and a vasculature of vessel network surrounding the periphery of tumorous tissue volume in which vessels tend to be sources of hot or cold spots (or stripes). HWH drafted the manuscript. TCS suggested some directions about the manuscript. TCS and CTL reviewed and analyzed mathematical models in the draft. All authors have read and approved the final manuscript.
